# Unveiling neural coupling within the sensorimotor system: directionality and nonlinearity

**DOI:** 10.1111/ejn.13692

**Published:** 2017-10-06

**Authors:** Yuan Yang, Julius P. A. Dewald, Frans C. T. van der Helm, Alfred C. Schouten

**Affiliations:** ^1^ Neuromuscular Control Laboratory Department of Biomechanical Engineering Delft University of Technology Delft The Netherlands; ^2^ Department of Physical Therapy and Human Movement Sciences Feinberg School of Medicine Northwestern University Chicago IL 60611 USA; ^3^ Department of Biomedical Engineering McCormick school of Engineering Northwestern University Evanston IL USA; ^4^ MIRA Institute for Biomedical Technology and Technical Medicine University of Twente Enschede The Netherlands

**Keywords:** corticomuscular interaction, cross‐frequency coupling, granger causality, sensorimotor system, sensory feedback

## Abstract

Neural coupling between the central nervous system and the periphery is essential for the neural control of movement. Corticomuscular coherence is a popular linear technique to assess synchronised oscillatory activity in the sensorimotor system. This oscillatory coupling originates from ascending somatosensory feedback and descending motor commands. However, corticomuscular coherence cannot separate this bidirectionality. Furthermore, the sensorimotor system is nonlinear, resulting in cross‐frequency coupling. Cross‐frequency oscillations cannot be assessed nor exploited by linear measures. Here, we emphasise the need of novel coupling measures, which provide directionality and acknowledge nonlinearity, to unveil neural coupling in the sensorimotor system. We highlight recent advances in the field and argue that assessing directionality and nonlinearity of neural coupling will break new ground in the study of the control of movement in healthy and neurologically impaired individuals.

## Introduction

The control of movement involves the interaction between the central nervous system and the periphery (Nielsen, 2016). Coupling of neural activity is thought to facilitate this large‐scale interaction in the nervous system (Varela *et al*., 2001). Synchronisation between the oscillatory activities of the sensorimotor cortex and the spinal motoneurone pool has been a focus of research in human motor control and has been introduced in clinical studies (see Salenius & Hari (2003) and Grosse *et al*. (2002) for reviews on the clinical applications). Corticomuscular coherence (CMC), that is coherence between scalp electroencephalography (EEG) and surface electromyography (EMG), is the most popular technique to quantify corticomuscular interactions (Mima & Hallett, 1999).

Voluntary movement control is initiated in the brain but also involves somatosensory feedback. Abundant evidence indicates that the oscillatory corticomuscular interactions originate not only from descending motor command but also is affected by ascending somatosensory feedback (Baker *et al*., 2003; Baker, 2007; Witham *et al*., 2011; Campfens *et al*., 2013, 2014). However, CMC cannot separate this bidirectionality in corticomuscular interaction; and more advanced measures are necessary to assess directionality (Witham *et al*., 2011; Campfens *et al*., 2014).

Many recent studies revealed the nonlinearity in the human sensorimotor system, especially the sensory pathways, resulting in neural coupling across frequencies (Snyder, 1992; Chen *et al*., 2010; Jamali & Ross, 2013). These cross‐frequency oscillations cannot be assessed nor exploited by linear measures such as CMC. To address these challenges, novel neural coupling measures have been proposed by our laboratory and other groups to provide directionality of information flow and acknowledge nonlinearity in the sensorimotor system. These studies provided new insights into the study of the control of movement in healthy and neurologically impaired individuals; however, an overview of these current advances is missing.

This review discusses potential drawbacks of using CMC (the linear coherence method) to assess corticomuscular interactions and then highlights recent innovations on directionality and nonlinearity in corticomuscular interactions. Previous reviews mainly focused on the functional meaning of CMC as well as its clinical applications (Hari & Salenius, 1999; Mima & Hallett, 1999; Grosse *et al*., 2002; Salenius & Hari, 2003; Baker, 2007; van Wijk *et al*., 2012). We argue that assessing directionality and nonlinearity of neural coupling will open new ground to understand neuronal communication between the central nervous system and the periphery during motor tasks.

## Corticomuscular coherence: what we know and what did we miss?

**Figure 1:**
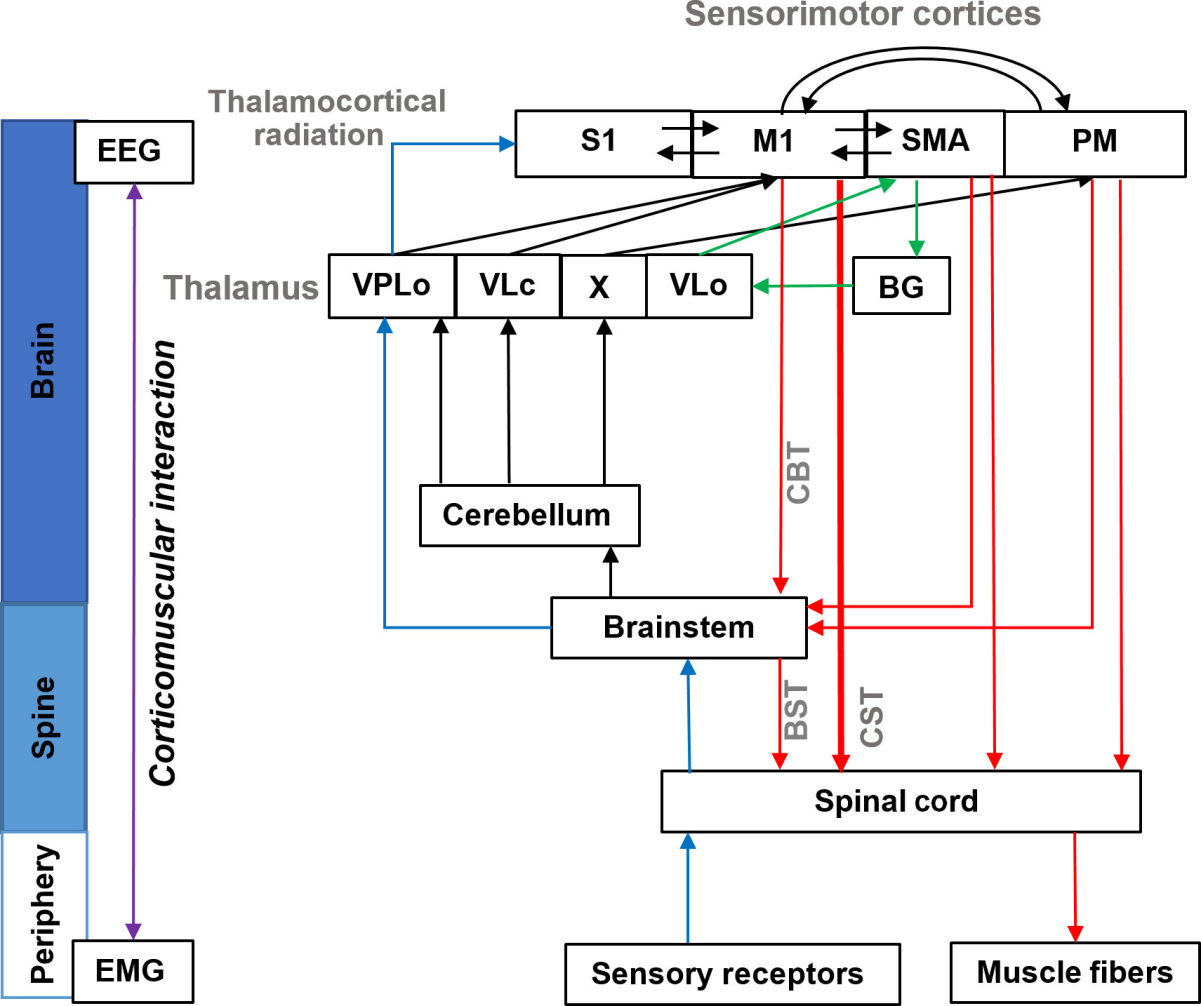
General overview of sensorimotor system. Sensorimotor control involves the periphery and various parts of the central nervous system including the sensorimotor cortices, basal ganglia, cerebellum, thalamus, brainstem and spinal cord (Kandel *et al*., 2012). Both descending motor output pathways (red lines) and ascending somatosensory feedback pathways (blue lines) can contribute to the corticomuscular coupling (Witham *et al*., 2011). Corticospinal tract (CST, bold red line) is the dominant, direct and fastest descending motor pathway in able‐bodied individuals (Lemon, 2008). In parallel with CST, there are multiple indirect pathways including corticobulbospinal pathways and CST tracts from secondary cortical motor cortices (SMA, premotor area) (Dum & Strick, 1991). Although contributions from indirect corticospinal pathways are relatively smaller compared to corticospinal tract in able‐bodied individuals, these indirect pathways may become more dominant when the corticospinal tract is damaged, e.g. after a stroke (Schwerin *et al*., 2008, 2011). Subcortical regions such as basal ganglia cerebellum and brainstem can also affect the corticomuscular coupling via the cortico‐subcortical loops (e.g. corticobasal ganglia loop indicated by green lines) and subcortical‐spinal tracts (Grosse *et al*., 2002; Salenius *et al*., 2002; Kelly & Strick, 2003; Akkal *et al*., 2007; Park *et al*., 2009; Airaksinen *et al*., 2015). S1, primary somatosensory cortex; M1, primary motor cortex; SMA, supplementary motor area; PM, premotor area; VPLo, oral portion of the ventral posterolateral nucleus; X, nucleus X; VL(o/c), oral/caudal portions of the ventral posterolateral nucleus; BG, basal ganglia; CBT, corticobulbar tract; BST, bulbospinal tract.

During a sustained muscle contraction, oscillatory activity of the motor cortex is typically shown in the alpha (~10 Hz) and beta (~20 Hz) band (Pfurtscheller & Da Silva, 1999). Although the information in both frequency bands can propagate to the periphery through the descending motor pathways (Baker *et al*., 2003), most studies reported only beta‐band CMC during sustained contractions. The beta‐band CMC is prominent during isometric motor tasks. The strength of beta‐band CMC is enhanced during a precise isometric motor action (Kristeva *et al*., 2007; Witte *et al*., 2007) and suppressed during a dynamic motor task (Kilner *et al*., 2003; Omlor *et al*., 2007). One popular opinion on CMC is that the beta‐band oscillatory synchronisation stabilises the steady descending motor output (Androulidakis *et al*., 2007). Jenkinson & Brown (2011) critically evaluated previous findings for and against this hypothesis and proposed that beta‐band oscillatory synchronisation is a measure reflecting the need of a new voluntary motor action during movement control. The voluntary motor action involves not only the cortical sensorimotor network and the spinal cord, but also subcortical neural networks such as cortical–subcortical loops involving basal ganglia and the cerebellum (Kelly & Strick, 2003; Akkal *et al*., 2007; Kandel *et al*., 2012). Thus, the value of CMC could be influenced by subcortical regions as well, although the coherence is measured between cortical oscillation and muscle activity (see Fig. 1).

Besides, several studies indicated that corticomuscular interaction is not mediated by the descending motor output alone. To test if the CMC is purely associated with the descending motor pathways, for example, Baker and Baker (2003) used benzodiazepine diazepam to enhance beta‐band oscillatory activity of the motor cortex. In their study, CMC did not increase in parallel with the ratio of EEG to EMG power, indicating that the CMC is not purely originated from descending signal propagation from the motor cortex to motor units. Their later studies focus on the relation between the coherence phase obtained by CMC and the transmission delay in the sensorimotor system. Riddle & Baker (2005) manipulated peripheral neural feedback loops by cooling the arm and found the ascending sensory feedback pathways can also alter corticomuscular coherence. Witham *et al*. (2011) used directed coherence to investigate the direction of oscillatory coupling between the brain and the muscle. These results further demonstrated that both descending motor output and ascending somatosensory feedback contribute to the CMC.

As shown in Fig. 1, the corticomuscular interaction could be influenced by various parts in the sensorimotor system. In the ascending pathway of the sensorimotor system, the somatosensory feedback during movement control is encoded by mechanoreceptors (i.e. muscle spindles and Golgi tendon organs), transmitted through synapses in the dorsal column nuclei at the brainstem [nucleus cuneatus (arm) and gracilis (leg)], and finally, reaches the somatosensory cortex via the thalamocortical somatosensory radiation. Many studies demonstrated the nonlinearity of the sensory ascending pathway. Early in 1992, Snyder and colleagues have found harmonic responses in the EEG when the participants received sinusoidal tactile vibrations in fingers and on the palm (Snyder, 1992). Similar results were also reported in later studies using magnetoencephalography (MEG) (Jamali & Ross, 2013). The nonlinearity of the ascending pathway could be originated from multiple sources in the periphery and the central nervous system. The mechanoreceptors such as muscle spindles are known to be highly nonlinear (Poppele, 1981). Rich nonlinearity has also been detected in the thalamocortical radiation and the corticocortical motor network (Chen *et al*., 2010; Langdon *et al*., 2011; Roberts & Robinson, 2012; Breakspear, 2017). A linear measure, as CMC, cannot assess the nonlinear interaction (Farina *et al*., 2014) and thus may miss connectivity characterising important neural coupling (Friston, 2001).

Furthermore, previous studies have reported large interindividual variance of CMC in healthy populations. For example, Ushiyama and colleagues measured CMC during isometric contraction of the tibialis anterior muscle on 100 healthy young individuals and found only 46 of 100 subjects showing significant CMC (Ushiyama *et al*., 2011). During upper limb motor tasks, significant CMC can be detected in around 80–90% healthy population (Mima & Hallett, 1999; Grosse *et al*., 2002; Kristeva *et al*., 2007; Mendez‐Balbuena *et al*., 2011; Witham *et al*., 2011; Campfens *et al*., 2013; von Carlowitz‐Ghori *et al*., 2015). However, testing on a small population, Mendez‐Balbuena and colleagues found large interindividual differences in the strength and bandwidth of CMC during low‐level static and dynamic forces generated by forearm muscles (Mendez‐Balbuena *et al*., 2011).

Despite these potential limitations, CMC has been applied in many clinical studies to investigate motor disorders. Modulation of the beta‐band CMC has been reported in individuals with Parkinson's disease and Parkinsonism (Caviness *et al*., 2003, 2006; Schnitzler *et al*., 2006; Hirschmann *et al*., 2013). In Parkinson's disease, synchronised beta‐band cortical oscillations are influenced by the dopamine level in the basal ganglia‐cortical motor loop (see the loop indicated by green lines in Fig. 1) (Jenkinson & Brown, 2011). Airaksinen *et al*. (2015) reported that the patients who have detectable beta‐band CMC usually have a better Unified Parkinson's Disease Score than the patients that do not. Furthermore, several studies showed that the beta‐band CMC which decreases in Parkinson's disease can be restored by therapeutic treatments with levodopa or deep brain stimulation to the subthalamic nucleus (Grosse *et al*., 2002; Salenius *et al*., 2002; Park *et al*., 2009). CMC has also been proposed as a measure to evaluate the motor recovery after stroke (Braun *et al*., 2007). As we indicated in Fig. 1, there are multiple indirect motor pathways (e.g. corticoreticulospinal pathways) in parallel with the direct corticospinal tract (Dum & Strick, 1991; Jang & Seo, 2014). Although contributions from these indirect motor pathways are relatively smaller compared to corticospinal tract (indicated by bold red line) in able‐bodied individuals, these indirect pathways may become more dominant when the corticospinal tract is damaged after a stroke (Schwerin *et al*., 2008, 2011). Such a change can prolong the time delay from the cortex to periphery during movement control (Meng *et al*., 2009) and modulate the value and frequency content of CMC (Yao & Dewald, 2006; von Carlowitz‐Ghori *et al*., 2014). Nevertheless, considering the large interindividual differences in a healthy population, the absence of CMC and modulation of CMC may not necessarily indicate pathological control of movement. This ambiguity could then lead to a challenge when using CMC in daily clinical practice. Moreover, several recent studies indicated the importance to assess the nonlinear neural coupling in motor disorder‐related diseases such as Parkinson's Disease (Cole & Voytek, 2017; Cole *et al*., 2017), which was neglected in the previous studies using CMC.

Thus, we argue that to develop a biomarker to distinguish pathological changes from normal variation in corticomuscular interaction we need measures which are not as variable as CMC. Several novel approaches are proposed in recent studies, showing the potential to address this need by acknowledging the closed‐loop and nonlinear properties of the sensorimotor system.

## Distinguishing the motor and somatosensory communications

Baker and colleagues revealed the complex nature of CMC with the bidirectional contributions from motor and somatosensory communications between the cortex and the periphery (Witham *et al*., 2011). The descending motor pathway and ascending somatosensory feedback pathway form a closed loop in the sensorimotor system. Thus, both EEG and EMG signals are influenced by the descending motor output and ascending somatosensory feedback.

Granger causality is a statistical concept of ‘cause‐and‐effect’ relation based on prediction (Granger, 1969). According to this concept, if a signal x(t) ‘Granger‐causes’ a signal y(t), then combining the history of x(t) should provide a better prediction of y(t) than using the history of y(t) alone. Linear Granger causality measures such as directed transfer function (DTF) and partial directed coherence (PDC) (Kaminski & Blinowska, 1991; Sameshima & Baccalá, 1999; Bastos & Schoffelen, 2015) are popular methods used in this closed‐loop problem to separate the descending connectivity from the cortex to the muscle and the ascending from the muscle to the cortex as well as their time delays (Meng *et al*., 2008; Witham *et al*., 2011).

**Figure 2:**
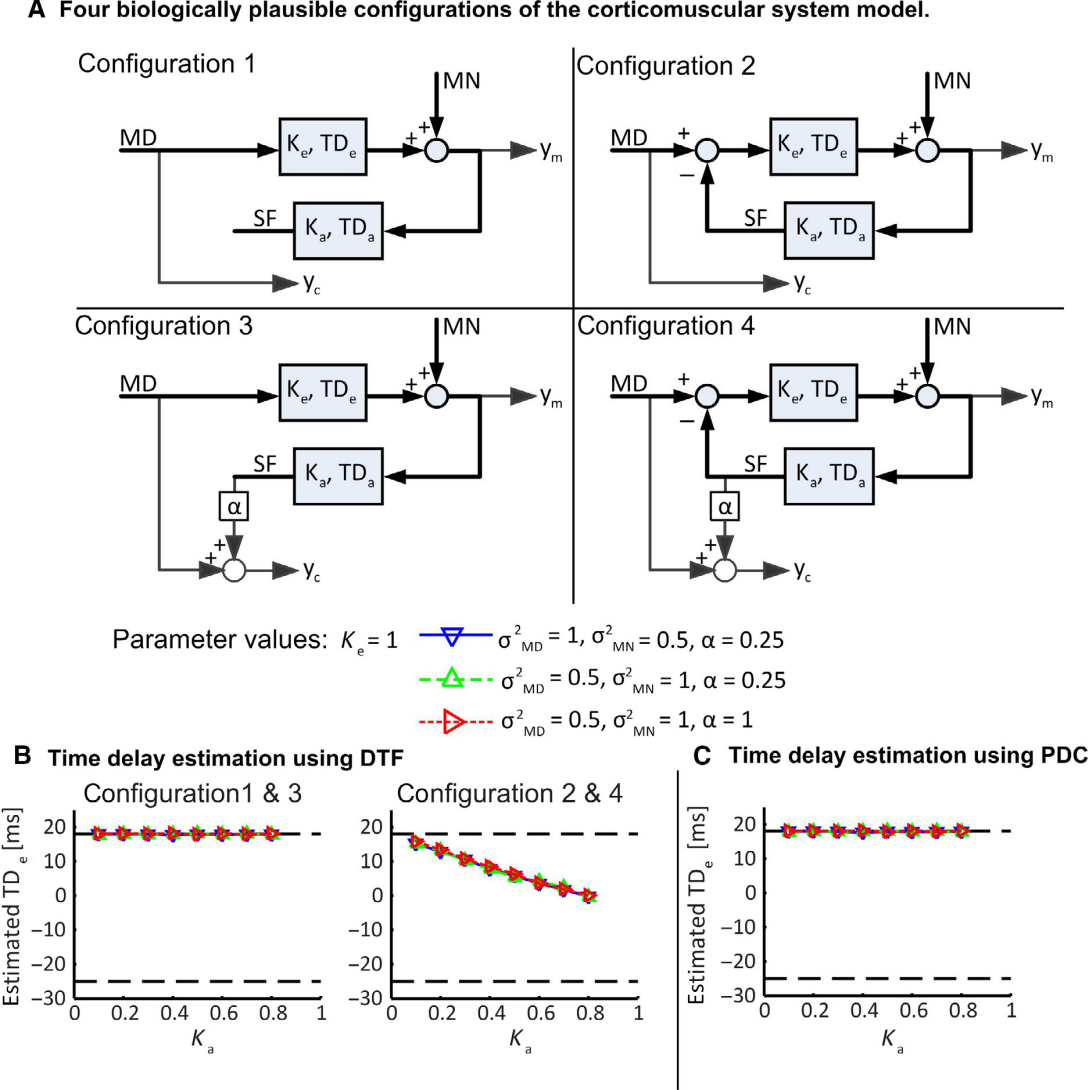
Time delay estimation in the corticomuscular system using two popular linear Granger causality measures: DTF and PDC. (A) Four biological plausible configurations of the corticomuscular system model. Campfens *et al*. (2014) modelled the corticomuscular system as a feedback system with cortical motor drive and motor noise. The descending motor pathway and ascending somatosensory feedback pathway were modelled with a gain (K) and a time delay (TD): 18 ms for descending pathway, 25 ms for ascending pathway based on experimental results from Rothwell *et al*. (1991) and Abbruzzese *et al*. (1985) for wrist muscles. The model is driven by both motor drive (MD, its variance σMD) and motor noise (MN, its variance σMN) and generates two outputs: a cortical signal (yc) and a muscle signal (ym). In the configurations 1 and 2, the cortical signal (yc) reflects the cortical motor drive only, while in the configurations 3 and 4, the cortical signal (yc) is also affected by the sensory feedback from ym. In the configurations 1 and 3, the sensory feedback does not modulate the motor drive, while in the configurations 2 and 4, the sensory feedback changes the motor drive, giving a closed‐loop system. (B) Time delay estimation using DTF only obtains the correct time delay in the configurations 1 and 3, where the sensory feedback does not modulate the motor drive. (C) Time delay estimation using PDC leads to the correct estimation in all configurations. The figure is reproduced, with permission, from Campfens *et al*. (2014) published in Journal of Computational Neuroscience.

Linear Granger causality measures are based on multivariate autoregressive (MVAR) modelling of stochastic processes which allows disentangling of the ‘cause‐and‐effect’ relation within a closed loop. However, the phase of the directional coherence presents the relative timing between neural signals within the closed loop and affected by an unknown external noise source (Schouten & Campfens, 2012). This effect may lead to the difference between estimated delays by Granger causality measures and the response latencies measured from experimental stimulation (i.e. transcranial magnetic stimulation and periphery nerve stimulation). Model simulations show only PDC could lead to the correct estimation of descending transmission delay (see Fig. 2) when sensory feedback modulates the cortical motor drive in a closed loop (Campfens *et al*., 2014).

Our group proposed to apply an external mechanical perturbation to the periphery for quantifying connectivity in the ascending somatosensory pathway (Campfens *et al*., 2013). The perturbation signal was imposed by an external mechanical source outside the physiological loop of sensorimotor system. Coherence between the perturbation and EEG (perturbation‐cortical coherence, PCC) represents unidirectional causality from the periphery to the cortex. In contrast to CMC which was not detectable in some subjects (Mendez‐Balbuena *et al*., 2011), significant PCC was found in all subjects in these experiments (Campfens *et al*., 2013). Later on, PCC was employed to evaluate ascending somatosensory pathway information transfer in the subacute stroke patients. These results suggested that motor function impairment may company with reduced somatosensory processing after stroke (Campfens *et al*., 2015). Previous studies have indicated that the importance of sensory feedback for motor recovery after stroke (Hamdy *et al*., 1998; Rossini *et al*., 2003). Thus, this new approach (i.e. PCC and mechanical perturbations) may provide an increased insight into mechanism of motor recovery in the subacute phase of stroke.

## Assessing the nonlinear corticomuscular interaction

Neural coupling measures based on surface recordings like EEG and EMG capture dynamic activities of underlying neural populations. The behaviour of a single neurone is highly nonlinear, showing a step‐like ‘all‐or‐nothing’ firing response. However, the behaviour of neurones in a population is typically rather similar. Therefore, nonlinear response properties of a single neurone could be smoothed by distribution of membrane thresholds across a population of neurones. This smoothing effect increases with the scale of the population, which could result in similar mean firing rates in neuronal populations with similar cell types and configurations (Breakspear, 2011). Thus, the communication between neuronal populations at the same frequency (iso‐frequency coupling) can be assessed by linear approaches such as coherence and cross‐correlation, even though the underlying neural elements are nonlinear.

Based on this hypothesis, linear approaches have been extensively used to investigate the connectivity between brain areas and the connectivity between the central nervous system and the periphery within the sensorimotor system (van Wijk *et al*., 2012). However, it is not clear how much information is missing when using this approach. When one uses a coherence measure to investigate the neural communication, the coupling across frequencies is ignored, especially between the neuronal populations which have very different mean firing rates such as the central nervous system and the periphery.

Several novel methods have been proposed to quantify nonlinear interactions in the sensorimotor system including both time and frequency domain approaches. Time domain methods are mainly based on mutual information (MI). In contrast to cross‐correlation reflecting *only* linear interactions, mutual information captures both linear and nonlinear relations between time series based on their statistical dependencies. In motor disorder studies, MI has been applied to assess functional connectivity between muscles (Madeleine *et al*., 2011, 2016). Similar to cross‐correlation/coherence, MI is a symmetric measure which cannot indicate the direction of information flow. Assuming x(t) and y(t) are two time series, then there is MI (x(t),y(t)) = MI (y(t),x(t)). By adding a time delay parameter in either of the signal, one can obtain a modulated, asymmetric manual information namely time‐delayed mutual information (TDMI), where TDMI (x(t), y(t+τ))  ≠ TDMI (y(t), x(t+τ)). TDMI has been proposed as a promising tool to investigate nonlinear corticomuscular interactions (Jin *et al*., 2010). However, as a time domain method, TDMI has limitations to reflect detail frequency contents of corticomuscular interactions. Moreover, this method requires long stationary time series (Jin *et al*., 2010) which could be a bottleneck in EEG experiments due to the nonstationary property of EEG signal (Zhan *et al*., 2006).

Recently, we proposed a frequency domain approach namely cross‐spectral coherence (CSC) to investigate the nonlinear corticomuscular interaction (Yang *et al*., 2016a). CSC is a generalised coherence framework for quantifying nonlinear coupling between signals across different frequency bands as well as the linear coupling within the same frequency band (Yang *et al*., 2016b). CSC is different from the generalised phase synchronisation index (Breakspear, 2002) which assesses the cross‐frequency coupling independent from the signal amplitude. CSC incorporates both phase and amplitude relations between signals, and its linear part is in line with the coherence measure used in CMC. A recent study found that oscillation activities of the motor cortex can transmit not only the phase but also amplitude dynamics through corticospinal tract (Bayraktaroglu *et al*., 2013). Thus, CSC is more suitable to assess nonlinear corticomuscular interactions compared to other phase synchronisation measures.

**Figure 3:**
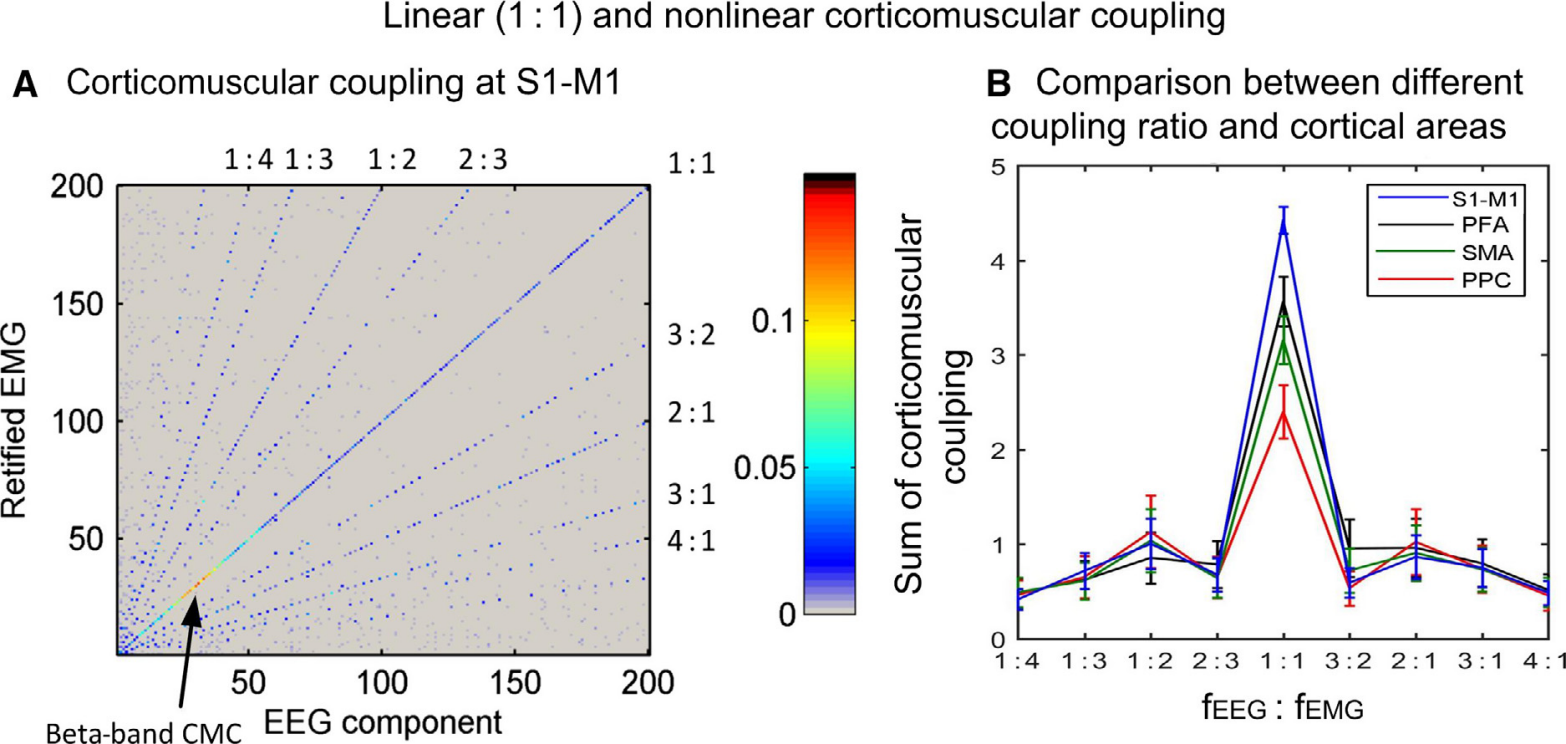
Linear and nonlinear corticomuscular coupling in healthy participants during an isotonic wrist flexion task. (A) Both linear (1 : 1) and nonlinear (n : m, n ≠ m) coupling are detected. Linear coupling shows a peak in the beta‐band. (B) Comparison of corticomuscular coupling between different coupling ratio and cortical areas. The linear coupling significantly reduced from the motor‐related cortical areas to sensory‐related areas, while no significant differences between different cortical areas are detected for nonlinear coupling. Due to the limited spatial resolution of EEG, the activities from S1 and M1 cannot be separated. The figure is reproduced, with permission, from Yang *et al*. (2016a) published in Frontiers in Computational Neuroscience.

Using CSC and independent component analysis, we assessed both linear and nonlinear interaction between muscle activity and multiple brain sources in healthy participants during an isotonic wrist flexion task (Yang *et al*., 2016a). In consistent with previous studies, we found beta‐band peak in the linear corticomuscular interaction for both motor and sensory‐related cortices, that is primary sensorimotor areas (S1‐M1), premotor area (PMA), supplementary motor area (SMA) and posterior parietal cortex (PPC). The magnitude of the beta‐band peak reduces from the motor‐related cortices to the sensory‐related cortices (see Fig. 3). These results indicate the beta‐band peak of CMC is more related to the motor output, although both the motor and somatosensory communications can contribute to CMC. This indication is supported by a case report showing the beta‐band CMC remains on a deafferented patient (Patino *et al*., 2008). Through theoretical derivation and experimental recording, Negro & Farina (2011b) demonstrated that cortical input can transmitted in a linear way to the motoneurone pool through the corticospinal tract. This direct, monosynaptic transmission of neural oscillations from the motor cortex to the motoneurone pool is thought to allow an efficient control of the muscle force output. Their simulations showed that the linear transmission becomes superior to the nonlinear transmission when more than four motoneurones are activated. Nevertheless, this transmission could be influenced by the nonlinear nature of the spiking processes of the active motoneurones, resulting in the modulation of CMC (Negro & Farina, 2011a). Furthermore, the corticospinal tract is not the only pathways that deliver common synaptic inputs to the motoneurone pool. Synaptic inputs from other pathways, such as reticulospinal, rubrospinal and vestibulospinal tracts (Clair, 2010) as well as monosynaptic sensory feedback in the spinal level (Nielsen, 2016), projected commonly to the motoneurone may decorrelate the output of the motoneurone pool with respect to the inputs from the cortex (Negro & Farina, 2011a) and even induce asynchronous coupling between the inputs and the output (Renart *et al*., 2010). A key distinction between synchronous and asynchronous coupling is that the former is essentially a linear transmission from the input to the output and the latter is highly nonlinear (Friston, 2001). Thus, investigating the modulation of linear coupling as well as the nonlinear coupling between the central nervous system and the motoneurone pool may provide useful information for assessing pathological conditions, such as stroke.

In the study of Yang *et al*. (2016a), we detected both harmonic and noninteger 2 : 3 coupling between muscle activity and cortical sources in healthy participants (see Fig. 3). Harmonic coupling with integer multiples of the stimulation frequencies (e.g. 2f, 3f, …) has been widely reported in cortical responses to somatosensory stimuli (Snyder, 1992; Jamali & Ross, 2013). Langdon and colleagues have reported noninteger coupling with the ratio 2 : 3 of brain response to fingertip stimulation (Langdon *et al*., 2011). A recent study from our group demonstrated that more than 80% of cortical response to the somatosensory input is generated from nonlinear neuronal interactions, resulting in cross‐frequency coupling with different coupling ratios (Vlaar *et al*., 2017). Computational studies based on neural mass/field models have demonstrated the nonlinear dynamics of sensory pathway, showing the same nonlinear ratios as we detected in the nonlinear corticomuscular interaction (Herrmann *et al*., 2016). Thus, a plausible hypothesis could be that the nonlinear corticomuscular interaction is mainly generated in the ascending somatosensory feedback pathways, although some contributions from the descending motor output pathways discussed above cannot be excluded.

**Figure 4:**
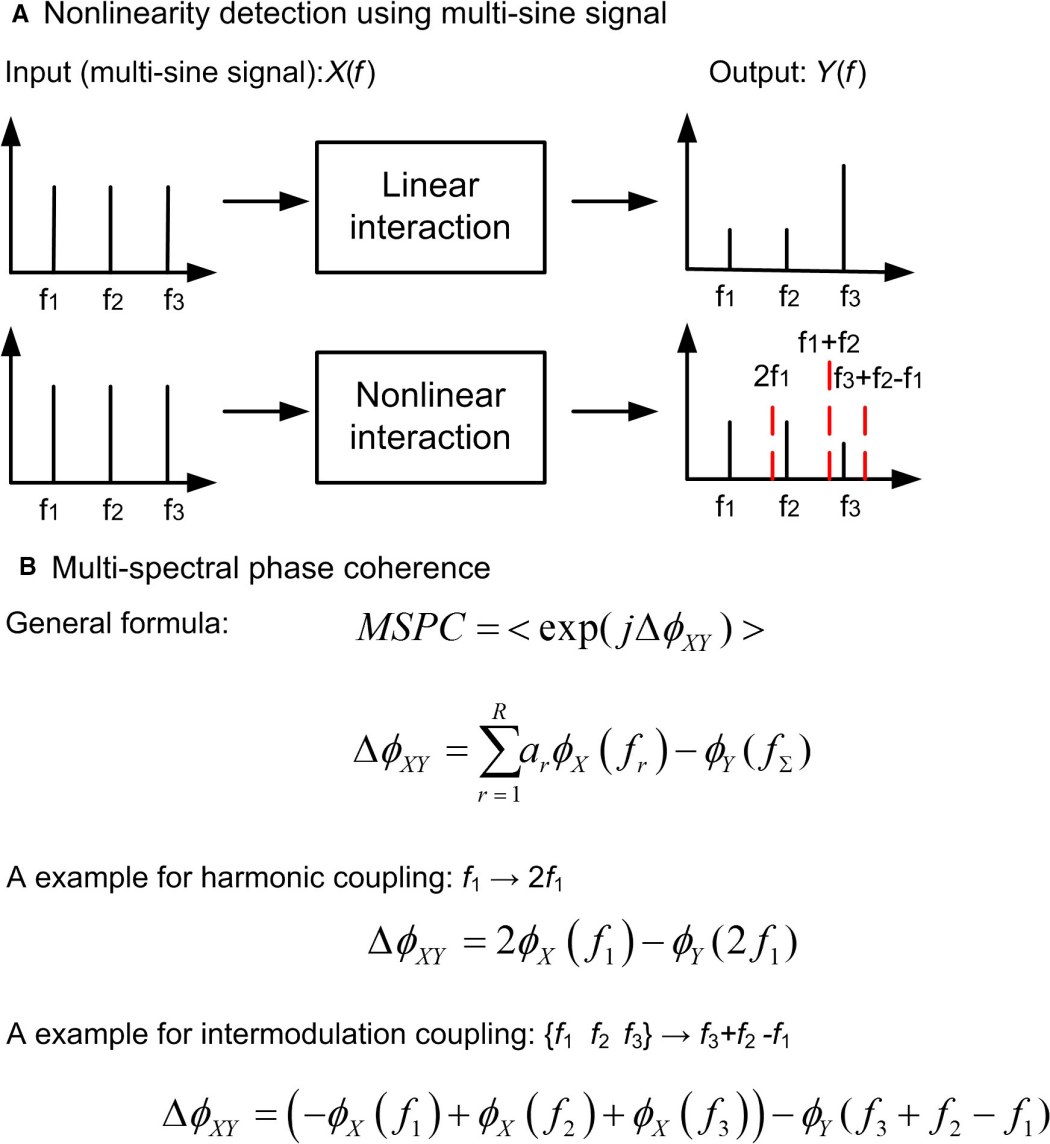
Multi‐sine signal and multi‐spectral phase coherence are key combinatorial approach to investigate nonlinear coupling. (A) Nonlinearity detection using multi‐sine signal. A linear interaction only generates the output at the same frequencies as the input (solid lines), while a nonlinear interaction can yield output spectral components at nonstimulated frequencies (dashed lines), such as the harmonic (e.g. 2f1) and intermodulation (e.g. f1 + f2, f3 + f2 − f1) frequencies. (B) The mathematical description of multi‐spectral phase coherence, which quantifies nonlinear coupling based on the phase difference between input and output components across frequencies. Examples of harmonic and intermodulation coupling are demonstrated. More details are available in Yang *et al*. (2016c).

Nevertheless, EEG and EMG are still within in a closed loop, which makes it difficult to distinguish the motor and sensory contributions (Schouten & Campfens, 2012). The application of external perturbations could be a promising way to ‘open’ the loop. Considering the nonlinearity of sensorimotor system, a periodic perturbation signal can be used. A periodic multi‐sine (sum‐of‐sinusoidal) signal contains only a limited number of sinusoids with carefully selected frequencies, leaving most frequency lines in the power spectrum ‘open’ for detecting and assessing nonlinearity (see Fig. 4A) (Pintelon & Schoukens, 2012). For example in the experiment of Yang *et al*. (2016d), the participants performed an isotonic wrist flexion torque when they were receiving the multi‐sine signal. The multi‐sine signal serves as the independent reference signal to infer the cause‐and‐effect relation in the sensorimotor loop as well as the nonlinearity.

To quantify nonlinear coupling and time delay in the sensorimotor system, we proposed multi‐spectral phase coherence (MSPC) method (Yang *et al*., 2016c). This method is capable of quantifying both harmonic and intermodulation nonlinear coupling from multiple input frequencies to the output frequencies (see Fig. 4B). Thus, MSPC can detect rich nonlinear interactions in the neural systems. The time delay between the input and the output can also be estimated based on the relative phase [see Yang *et al*. (2016c) for details]. The time delay in the ascending somatosensory pathway assessed by the multi‐sine perturbation and MSPC is line with the latency of dominant somatosensory evoke potential (P45/P50) caused by mechanical perturbations. Assessing the directional nonlinear corticomuscular interaction in the perturbed task, we found that the directional nonlinear connectivity from the brain to the muscle is very weak (Yang *et al*., 2016d). This result confirmed the hypothesis that nonlinear corticomuscular coupling is mainly originated from ascending somatosensory pathways instead of descending motor pathways. Previous studies have revealed the nonlinear dynamics of muscle spindles (Kearney & Hunter, 1988) and the thalamocortical radiation (Langdon *et al*., 2011; Roberts & Robinson, 2012). Thus, the nonlinearity in the ascending somatosensory can come from both the central nervous system and the periphery. A recent study indicated that motor disorders such as essential tremor can result in the abnormality of nonlinear dynamics in the thalamocortical radiation (He *et al*., 2016), indicating clinical values of assessing nonlinear interaction in the sensorimotor system.

Finally, Cole & Voytek (2017) very recently highlighted that neural oscillations can have nonsinusoidal shapes of waveform, showing complicated nonlinear dynamics, which are often overlooked in the studies using Fourier spectral‐based nonlinear analysis. Their results indicated that this nonsinusoidal oscillation and nonlinear dynamics may be related to cortical pathophysiology in Parkinson's Disease (Cole *et al*., 2017). As nonsinusoidal features of neural oscillations cannot be fully captured using a Fourier transform, a few possible solutions such as using a matching pursuit algorithm or empirical model decomposition were discussed in the review article by Cole & Voytek (2017). A combination of non‐Fourier transform‐based decomposition with nonlinear neural analysis could be very promising for investigating complicated nonlinear dynamics and underlying physiological mechanism, especially for movement disorder‐related diseases such as Parkinson's disease.

## Conclusion

Many studies have focused on exploring functional roles of corticomuscular coherence, providing evidence on the putative functional significance and clinical relevance of neural coupling measures. Nevertheless, numerous questions have also been proposed about what information can be learnt and what information should be ignored by investigating corticomuscular coherence. Challenges partially come from limitations of corticomuscular coherence being a linear and nondirectional mathematical technique. In this review, we argue that novel methods are needed (i) to distinguish motor and somatosensory communications in the sensorimotor system; and (ii) to exploit the nonlinear nature of neural coupling between the central nervous system and the periphery. Applying these new approaches may eventually provide a more detailed mechanistic understanding of neural communications during movement control and lead to useful tools for studying motor disorder‐related diseases such as stroke and Parkinson's disease.

## Conflict of interest

Authors declare that they do not have any conflict of interests.

## Author contributions

YY and AS drafted and wrote the manuscript. JD and FH commented and revised the manuscript.
