# Supplementary material for: Unveiling neural coupling within the sensorimotor system: directionality and nonlinearity
Source: Eur J Neurosci. 2017 Oct 6;48(7):2407–15. doi: 10.1111/ejn.13692 (PMC6221113; doi:10.1111/ejn.13692)
Supplement: Supplementary file 1 [file EJN-48-2407-s001.pdf]

EJN European Journal of Neuroscience FENS Federation of European Neuroscience Societies

Unveiling neural coupling within the sensorimotor system: directionality and nonlinearity

Yuan Yang, Julius P. A. Dewald, Frans C. T. van der Helm & Alfred C. Schouten

Review timeline:

Submission date:	16 June 2017
Editorial Decision:	28 July 2017
Revision received:	18 August 2017
Accepted:	05 September 2017

Editor: Ali Mazaheri

1st Editorial Decision 28 July 2017

Dear Dr. Yang,

Your manuscript has been reviewed by external reviewers as well as by the Section Editor, Dr. Ali Mazaheri, and ourselves. The reviewers appreciated the timeliness and relevance of your review and are supportive of its publication in EJN. Both however, raised a couple of minor points that need to be addressed. In particular, the clarification of Figure 4 and clarification/mention of the importance/role of the basal ganglia and possibly other sub-cortical structures; they are part of the sensorimotor system and contribute to aberrant oscillations in disease.

In your revised submission, please also address the following points that we noted.
- Please remove section numbering
- Please include Author contributions
- Please ensure that you have permission to reproduce figures and provide details.

If you are able to respond fully to the points raised, we would be pleased to receive a revision of your paper within 30 days.

Thank you for submitting your work to EJN.

Kind regards,

Paul Bolam & John Foxe
co-Editors in Chief, EJN

Reviews:

Reviewer: 1 (Franca Tecchio, Institute of Cognitive Sciences and Technologies, Italy)

Comments to the Author
The review relates a definitely relevant subject, which is the proper mathematical tools to assess the synchronization phenomena within the sensorimotor system expressing the motor commands. I also definitely agree about the need to take into account linear and nonlinear phenomena, and the Authors propose some solutions in this direction. In particular, I consider pertinent and clever to couple mathematical and experimental approaches, as the Authors do. During an isometric wrist contraction, they apply an external mechanical perturbation to the periphery for quantifying connectivity in the ascending somatosensory pathway as a way to “open” the sensorimotor loop. It remains to be further investigated whether the assessment of the afferent sensory counterpart depends on the kind of interfering external mechanical perturbation. In particular, application of sinusoidal perturbations leads to no surprisingly higher sinusoidal coherence (perturbation-cortical coherence, PCC) than the physiological signals coordinating movements (CMC).
I only have some notes and integrations to suggest:
- Correct the indication of ‘CMC which is only shown in 40-50% healthy population’ and either

integrate or remove the reported paper Ushiyama et al., 2011, which does not relate to the upper limb. The overall experience indicates for the hand movements CMCs above confidence limits in about 80-90% of healthy people (works of Kristeva group's, Brawn, Curio, Mima…)
- Figure 4 is not really informative in this form: either better elucidate its meaning or omit it in the review.

Reviewer: 2 (Dora Hermes, Stanford University, USA)

Comments to the Author
In this special issue review Yang and Schouten provide an overview of the current advances in the non-linear analyses of corticomuscular interaction. They describe the current research in corticomuscular coherence, distinguishing between motor and somatosensory communications and assessing nonlinear corticomuscular interactions. It is a very focused and concise paper and it is clearly written. I only have a few questions.

One of the main conclusions of the paper is that they discuss the useful tools to study movement related disorders, such as Parkinson's disease. However, Parkinson's is a disease in which subcortical areas play a large role. Also, the title is about neural coupling in 'the sensorimotor system', which (in my view) includes the basal ganglia. Similarly, the discussed beta oscillations are thought to be highly influenced by subcortical-cortical interactions. In this review the basal ganglia are left completely out of view. Can the authors clarify? Maybe an overview figure of the anatomical sensorimotor system and the aspects discussed by the authors would be helpful here?

Very recent work by Voytek has highlighted that oscillations can have a non-sinusoidal shape (Cole and Voytek, 2017 TICS). They also studied this in Parkinson's disease (Cole, …, Voytek, 2017 Journal of Neuroscience). Maybe this is another type of non-linearity upon which the authors could briefly touch and discuss how to take this into account in the other methods?

In the introduction the review states that it will discuss the controversial opinions. In the paper it is not quite clear which aspects are controversial.

There is a typo at the end of section 3 (i.e. PPC and mechanical…) should be (i.e. PCC and mechanical).

Authors' Response 18 August 2017

We would like to thank the editors and external reviewers for their useful comments and suggestions. Their comments clearly helped us improve our manuscript. We anticipate that the revised version addresses all issues. Modifications to the manuscript are underlined in the main document. The answers to the reviewers' comments are included below in *italics*. All references given in this letter can also be found in the reference list of the revised manuscript.

Response to Editors

Your manuscript has been reviewed by external reviewers as well as by the Section Editor, Dr. Ali Mazaheri, and ourselves. The reviewers appreciated the timeliness and relevance of your review and are supportive of its publication in EJN.

We are glad that the contributions of our work were recognized by the editors and external reviewers. We appreciate the comments, and present our reply to each specific comment below.

Both however, raised a couple of minor points that need to be addressed. In particular, the clarification of Figure 4 and clarification/mention of the importance/role of the basal ganglia and possibly other sub-cortical structures; they are part of the sensorimotor system and contribute to aberrant oscillations in disease.

We revised the manuscript based on the review comments. According to the suggestion from Reviewer 1, we removed the original Figure 4, because the figure itself is not informative and the content has been provided in the text of revised manuscript. We now mention the important role of the basal ganglia as well as other subcortical structures, and explained how they may contribute to aberrant oscillations in motor disorders (e.g. Parkinson's Disease and stroke, see modifications in Pages 5-6). Furthermore, we added a new figure (Figure 1 in the revised manuscript) to give the general overview of sensorimotor system as Reviewer 2 suggested.

In your revised submission, please also address the flowing points that we noted.
- Please remove section numbering
- Please include Author contributions
- Please ensure that you have permission to reproduce figures and provide details.

Thank you for the comments.

- *We removed section numbering in the revised manuscript.*
- *We added the Author Contributions section (see Page 15). Prof. Dr. Julius P.A. Dewald and Prof. Dr. Frans C.T. van der Helm made significant contributions to this review article during the revision, especially for the part related to clinical impact, neurophysiology and motor control relevant sections of the revised manuscript. We therefore added them as co-authors and highlighted their contributions in the Author Contribution section.*
- *We have got the permissions from other publishers/authors to reproduce figures. Details provided below:*
  - *Figure 1 is a new figure.*
  - *Figure 2 is reproduced from Campfens (2014) published in Journal of Computational Neuroscience by Springer Nature. We have got the permission from the publisher to reproduce this figure (license number: 4160581248083, Copyright Clearance Center). The license is provided as a separate file. We mentioned the re-production and cited Campfens (2014) in the caption of this figure.*
  - *Figure 3 is reproduced from our own work Yang et al (2016a) published in the Frontiers in Computational Neuroscience. This is an open-access publication under the CC-BY license, which permits unrestricted use, distribution, and reproduction in any medium, provided the original authors and the source are credited. We confirmed with the management team of Frontiers in Computational Neuroscience that no additional license is needed once we cite the paper and mentioned the journal in the citation. Thus, we mentioned the re-production and cited Yang et al (2016a) in the caption of this figure.*
  - *Figure 4 is a figure generated based on the theoretical contents of Yang et al (2016c). Please note that this figure is not a reproduction of an existing figure in a published paper.*

Responses to Reviews:

Reviewer: 1

The review relates a definitely relevant subject, which is the proper mathematical tools to assess the synchronization phenomena within the sensorimotor system expressing the motor commands. I also definitely agree about the need to take into account linear and nonlinear phenomena, and the Authors propose some solutions in this direction. In particular, I consider pertinent and clever to couple mathematical and experimental approaches, as the Authors do. During an isometric wrist contraction, they apply an external mechanical perturbation to the periphery for quantifying connectivity in the ascending somatosensory pathway as a way to “open” the sensorimotor loop. It remains to be further investigated whether the assessment of the afferent sensory counterpart depends on the kind of interfering external mechanical perturbation. In particular, application of sinusoidal perturbations leads to no surprisingly higher sinusoidal coherence (perturbation-cortical coherence, PCC) than the physiological signals coordinating movements (CMC).

Thank you for the comments. We are happy that the contribution of our work is recognized by the reviewer. We appreciate the comments, and present our reply to each of your specific comments below.

I only have some notes and integrations to suggest:

- Correct the indication of ‘CMC which is only shown in 40-50% healthy population’ and either integrate or remove the reported paper Ushiyama et al., 2011, which does not relate to the upper limb. The overall experience indicates for the hand movements CMCs above confidence limits in about 80-90% of healthy people (works of Kristeva group’s, Brawn, Curio, Mima…)

  Thank you for the suggestion. We have corrected this indication by clearly mentioning that 1) the finding from Ushiyama is in the tibialis anterior muscle and 2) for upper limb, the significant CMC can be detected in around 80-90% healthy subjects.

Changes in text are provided below and underlined in the manuscript (see Page 5):
"…For example, Ushiyama and colleagues measured CMC during isometric contraction of the tibialis anterior muscle on 100 healthy young individuals and found only 46 out of 100 subjects showing significant CMC (Ushiyama et al., 2011). During upper limb motor tasks, significant CMC can be detected in around 80-90% healthy population (Mima & Hallett, 1999; Grosse et al., 2002; Kristeva et al., 2007; Mendez-Balbuena et al., 2011; Witham et al., 2011; Campfens et al., 2013; von Carlowitz-Ghori et al., 2015)…."

and Page 8:
"…In contrast to CMC which was not detectable in some subjects (Mendez-Balbuena et al., 2011), significant PCC was found in all subjects in these experiments (Campfens et al., 2013) …"

- Figure 4 is not really informative in this form: either better elucidate its meaning or omit it in the review.

  Thank you for the suggestion. We agree this figure itself is not really informative. Consequently, we followed your suggestion and remove it and provided the associated content in text only. Please see the changes in Page 13: "…Assessing the directional nonlinear corticomuscular interaction in the perturbed task, we found that directional nonlinear connectivity from the brain to the muscle is very weak (Yang et al., 2016d). This result confirmed the hypothesis that nonlinear corticomuscular coupling is mainly originated from ascending somatosensory pathways instead of descending motor pathways."

Reviewer: 2

Comments to the Author
In this special issue review Yang and Schouten provide an overview of the current advances in the non-linear analyses of corticomuscular interaction. They describe the current research in corticomuscular coherence, distinguishing between motor and somatosensory communications and assessing nonlinear corticomuscular interactions. It is a very focused and concise paper and it is clearly written. I only have a few questions.

Thank you for the comments. We appreciate the comments, and present our reply to each of your specific comments below.

- One of the main conclusions of the paper is that they discuss the useful tools to study movement related disorders, such as Parkinson's disease. However, Parkinson's is a disease in which subcortical areas play a large role. Also, the title is about neural coupling in 'the sensorimotor system', which (in my view) includes the basal ganglia. Similarly, the discussed beta oscillations are thought to be highly influenced by subcortical-cortical interactions. In this review the basal ganglia are left completely out of view. Can the authors clarify? Maybe an overview figure of the anatomical sensorimotor system and the aspects discussed by the authors would be helpful here?

  Thank you for the comment. We agree that subcortical areas, especially basal ganglia, which play an important role in Parkinson's Disease, and influence beta oscillations and CMC. We added a few discussion sentences to clarify this issue and provided an overview figure of sensorimotor system (see new Figure 1, generated based on the knowledge from the book "Principles of Neural Science" fifth edition, edited by Kandel et al in 2012) in the revised manuscript.

  Changes in text are provided below and underlined in the manuscript (see Page 4):
  *"…The voluntary motor action involves not only the cortical sensorimotor network and the spinal cord, but also sub-cortical neural networks such as cortical-subcortical loops involving basal ganglia and the cerebellum (Kelly & Strick, 2003; Akkal et al., 2007; Kandel et al., 2012). Thus, the value of CMC could be influenced by subcortical regions as well, though the coherence is measured between cortical oscillation and muscle activity (see **Figure 1**)"*

  "As shown in Figure 1, the corticomuscular interaction could be influenced by various components of the sensorimotor system…"

  and the end of Page 5 to Page 6:

"… In Parkinson's disease, synchronised beta band cortical oscillations are influenced by the dopamine level in the basal ganglia-cortical motor loop (see the loop indicated by green lines in ***Figure 1****) (Jenkinson & Brown, 2011). Airaksinen et al. (2015) reported that the patients who have detectable beta band CMC usually have a better Unified Parkinson's Disease Score than the patients that does not. Furthermore, several studies showed that the beta band CMC which decreases in Parkinson's disease can be restored by therapeutic treatments with levodopa or deep brain stimulation to the subthalamic nucleus (Grosse et al., 2002; Salenius et al., 2002; Park et al., 2009) …"*

- Very recent work by Voytek has highlighted that oscillations can have a non-sinusoidal shape (Cole and Voytek, 2017 TICS). They also studied this in Parkinson's disease (Cole, …, Voytek, 2017 Journal of Neuroscience). Maybe this is another type of non-linearity upon which the authors could briefly touch and discuss how to take this into account in the other methods?

  Thank you for these interesting references. We have read the papers and found the studies very relevant to the topic discussed in our manuscript. We therefore added a paragraph on Pages 13-14 to highlight their findings and mentioned non-Fourier transform based nonlinear analysis could be very promising to investigate complicated nonlinear dynamics, especially for pathophysiological studies. We copied this new paragraph from the revised manuscript below for your review:

  "Finally, Cole and Voytek (2017) very recently highlighted that neural oscillations can have non-sinusoidal shapes of waveform, showing complicated nonlinear dynamics, which are often overlooked in the studies using Fourier spectral based nonlinear analysis. Their results indicated that this non-sinusoidal oscillation and nonlinear dynamics may be related to cortical pathophysiology in Parkinson's Disease (Cole et al., 2017). Since non-sinusoidal features of neural oscillations cannot be fully captured using a Fourier transform, a few possible solutions such as using a matching pursuit algorithm or empirical model decomposition were discussed in the review article by Cole and Voytek (2017). A combination of non-Fourier transform based decomposition with nonlinear neural analysis could be very promising for investigating complicated nonlinear dynamics and underlying physiological mechanism, especially for movement disorder related diseases such as Parkinson's disease."

- In the introduction, the review states that it will discuss the controversial opinions. In the paper it is not quite clear which aspects are controversial.

  We apologize for this confusing statement. In this review article, we discussed the potential drawbacks of using linear coherence to assess corticomuscular interaction, rather than controversial opinions on corticomuscular coherence. We have corrected this statement in the revised manuscript (see Page 3) to "This review discusses potential drawbacks of using CMC (the linear coherence method) to assess corticomuscular interactions, and then highlights recent innovations on directionality and nonlinearity of corticomuscular interactions."

- There is a typo at the end of section 3 (i.e. PPC and mechanical…) should be (i.e. PCC and mechanical).

  Thank you for the comment. We corrected this typo in the revised manuscript (see Page 8).
